# Paradoxical PM2.5-longevity estimates in global country panels are sensitive to differential trend control: a specification analysis, 2000–2023

**DOI:** 10.3389/fpubh.2026.1936760

**Published:** 2026-10-01

**Authors:** Hongzhi Lu, Hongxue Lu

**Affiliations:** 1 School of Industrial Technology, Universiti Sains Malaysia, Gelugor, Malaysia; 2 Faculty of Education, University of Malaya, Kuala Lumpur, Malaysia

**Keywords:** ambient air pollution, differential trends, life expectancy, negative control, panel data, PM2.5, two-way fixed effects

## Abstract

**Background:**

Country-year panels are widely used for environmental health research, but heterogeneous national trajectories can produce ecological coefficients that conflict with established health evidence.

**Methods:**

We assembled 4,406 country-year observations from 186 economies in the World Bank World Development Indicators for 2000–2023. Annual life expectancy was regressed on population-weighted ambient PM2.5 exposure using economy and year fixed effects, with adjustment for gross domestic product per capita, health expenditure, and urbanization. The original sensitivity analyses examined alternative weights, covariance estimators, exposure lags, sample restrictions, and temporal transformations; a future-exposure negative control tested residual temporal structure. Economy-specific trends and rolling five-year differences were added after the conventional estimate showed an unexpected positive sign.

**Results:**

The conventional adjusted model associated a 10 μg/m^3^ higher PM2.5 exposure with 0.406 additional years of life expectancy (95% confidence interval, 0.144 to 0.668). The coefficient changed to −0.023 years (95% confidence interval, −0.175 to 0.129) after adding economy-specific linear trends and to −0.006 years (95% confidence interval, −0.085 to 0.073) in first differences. PM2.5 measured 2 years after the outcome was positively associated with current life expectancy in a separate-lead model (0.296 years; 95% confidence interval, 0.042 to 0.549), but the +2-year lead was attenuated and imprecise after adjustment for current exposure (0.152 years; 95% confidence interval, −0.039 to 0.342; *p* = 0.119). Regional estimates also differed in direction.

**Conclusion:**

The positive conventional coefficient was not robust to trend-focused specifications, and the future-exposure pattern raised concern about unresolved temporal structure without constituting a stand-alone falsification test. The pattern is consistent with unresolved differential trajectories, but the analysis does not establish that trend heterogeneity is the sole source of bias. Aggregate PM2.5-longevity studies require explicit estimand definition, trend diagnostics, change-based estimators, covariance sensitivity checks, and temporal negative controls before interpreting coefficients as health effects.

## 1 Introduction

Ambient fine particulate matter with an aerodynamic diameter no greater than 2.5 μm (PM2.5) is an established environmental health hazard. The World Health Organization (WHO) recommends an annual mean PM2.5 guideline value of 5 μg/m^3^ and concludes that long-term exposure contributes to premature mortality from cardiovascular, respiratory, and other causes (1). Nationally comparable PM2.5 exposure and life-expectancy indicators are now available through the World Bank World Development Indicators (WDI), enabling global longitudinal comparisons (2, 3). This accessibility creates an opportunity to study environmental and population-health trajectories, but it does not by itself make country-year variation a valid natural experiment.

Nitrogen dioxide (NO2) and other co-pollutants are also relevant to population health; long-term NO2 exposure has been associated with mortality in systematic-review evidence (4). This analysis nevertheless focused on PM2.5 because the research question arose from the originally selected, globally harmonized WDI PM2.5 series and because the study was designed as a specification analysis of one exposure-outcome pair, not as a comparative pollutant-burden analysis. Adding NO2 without a matched, defined exposure product and source-specific design could change geographic coverage, introduce co-pollutant collinearity, and create a different estimand. Multi-pollutant analyses using harmonized PM2.5, NO2, ozone, and source-composition data are therefore an important extension rather than a substitute for the present analysis.

Several research designs support a harmful relation between particulate exposure and longevity. In 211 US county units, a 10 μg/m^3^ decline in PM2.5 was associated with a 0.61-year increase in life expectancy after adjustment for socioeconomic, demographic, and smoking proxies (5). A regression-discontinuity analysis of China’s Huai River heating policy estimated that 10 μg/m^3^ higher PM10 reduced life expectancy by 0.64 years, with cardiorespiratory mortality accounting for most of the difference (6). Global exposure-response modeling has attributed substantial losses of life expectancy to ambient air pollution (7), and systematic reviews link long-term particulate exposure with higher all-cause and cause-specific mortality (8). The Global Exposure Mortality Model likewise integrates cohort evidence across a wide exposure range rather than inferring risk from national time trends alone (9). More recent reviews report consistent associations between traffic-related pollution and non-accidental mortality (10), between long-term particulate exposure and cardiorespiratory outcomes in low- and middle-income countries (11), and between ambient PM2.5 and multiple health outcomes in Asia-Pacific cohorts (12). Source-resolved European cohort analyses further show that mortality associations differ by particle source and composition (13).

Country and subnational panel studies occupy a different evidentiary position. A recent macroeconomic analysis of highly polluted countries reported lower life expectancy with greater air pollution after dynamic-panel adjustment (14). A US state panel found a negative PM2.5-life expectancy association that was stronger at higher income inequality (15). These aggregate studies can extend geographic coverage, yet their coefficients combine exposure measurement, demographic change, economic development, health-system change, migration, and other processes operating at the same temporal scale. Questions about the causal interpretation of regression coefficients in PM2.5 and life-expectancy analyses have therefore persisted, including sensitivity to influential observations, non-linear response, and residual temporal confounding (16).

The choice of fixed effects is especially consequential when exposure and outcome trends differ among places. A 2026 analysis of 52 two-way fixed-effects specifications for weekly PM2.5 and respiratory hospitalizations in California found meaningful changes in the target coefficient across spatial and temporal fixed-effect combinations (17). More generally, two-way fixed-effects regression removes time-invariant differences among units and shocks common to all units, but it does not automatically remove unit-specific trends or time-varying confounding (18, 19). Negative-control exposures can reveal residual bias when a variable that cannot plausibly cause an earlier outcome remains associated with that outcome (20, 21). External cohort, quasi-experimental, and burden-of-disease evidence establishes that particulate pollution is harmful at the population level (5–13, 22), but that evidence cannot make every ecological panel coefficient causally valid. If exposure measured after an outcome remains associated with that earlier outcome, the result raises concern about residual temporal structure. Because serially persistent exposure makes a single future value non-independent, temporal diagnosis is strengthened by distributed and conditional lead models; it should not be treated as a stand-alone corrected health-effect estimate (20, 21).

This study evaluated specification dependence in a global country-year PM2.5 and life-expectancy panel. Three questions were addressed. First, what association is produced by a conventional adjusted model containing country and year fixed effects? Second, does the coefficient persist under population weighting, alternative covariance estimators, exposure lags, country-specific trends, and change-based estimators? Third, does a future-exposure negative control or regional heterogeneity reveal residual structure incompatible with a health-effect interpretation? The objective was not to estimate an attributable burden or individual-level effect. It was to determine whether the aggregate coefficient remained directionally and temporally credible under explicit falsification and sensitivity tests.

## 2 Materials and methods

### 2.1 Study design and reporting

We conducted an ecological longitudinal specification analysis using annual national indicators. Reporting followed the Strengthening the Reporting of Observational Studies in Epidemiology (STROBE) recommendations where applicable to an aggregate panel study (23). The unit of analysis was an economy-year. All data were public, aggregated, and contained no individual records. The analysis cutoff was 31 December 2023, which was the latest year for which matched PM2.5 exposure and outcome data were available in the WDI release updated on 1 July 2026. Data were accessed and frozen on 13 July 2026.

The original pre-review analysis included a conventional two-way fixed-effects model, an unadjusted fixed-effects baseline, population weighting, clustered and Driscoll–Kraay covariance estimators, one- and two-year exposure lags, a panel-length restriction, a pre-2020 restriction, exposure winsorization, first differences, a two-year future-exposure negative control, and region-stratified models. The positive sign of the conventional estimate was unexpected because toxicologic, cohort, and quasi-experimental evidence predicts shorter, not longer, longevity at higher PM2.5 exposure; the sign discrepancy therefore triggered specification diagnostics rather than a protective interpretation. The 2000–2019 restriction was included to remove the acute pandemic mortality shock and contemporaneous changes in mobility, emissions, and health-service use from one sensitivity specification. It was not intended to imply that PM2.5 was unrelated to COVID-19 severity: ecological evidence has associated long-term PM2.5 exposure with higher COVID-19 mortality, while demographic studies document large pandemic-related life-expectancy losses (24–26). Country-specific linear trends and rolling five-year differences were then added as *post hoc* diagnostic specifications to distinguish a stable within-country association from dependence on low-frequency trajectories. All specifications, including null and sign-changing results, were retained.

### 2.2 Data sources and eligibility

Six WDI indicators were retrieved through the World Bank application programming interface for all non-aggregate economies for each year from 2000 through 2023: life expectancy at birth, total (SP.DYN.LE00.IN); PM2.5 air pollution, mean annual exposure (EN.ATM.PM25.MC.M3); gross domestic product (GDP) per capita based on purchasing power parity in constant 2021 international dollars (NY.GDP.PCAP.PP.KD); current health expenditure as a percentage of GDP (SH.XPD.CHEX.GD.ZS); urban population as a percentage of total population (SP.URB.TOTL.IN.ZS); and total population (SP.POP.TOTL). The PM2.5 indicator is a national population-weighted annual mean derived from satellite aerosol optical depth, chemical transport models, and available ground monitoring (2). The life-expectancy indicator is period life expectancy at birth, expressed in years (3).

The API returned 217 non-aggregate economies. An economy-year entered the analysis only when life expectancy, PM2.5, GDP per capita, health expenditure, and urbanization were all observed. No interpolation, extrapolation, carry-forward or backward filling, or model-based imputation was performed; an economy-year with any missing analysis variable was excluded from the complete-case model (27). The complete-case panel contained 4,406 observations from 186 economies; the median economy contributed 24 years, with a range of 11 to 24 years. Compared with the 186 included economies, the 31 excluded economies were more often high income (77.4% vs. 33.3%) and from Latin America & Caribbean (35.5% vs. 16.7%) or East Asia & Pacific (25.8% vs. 15.6%), and less often from Sub-Saharan Africa (6.5% vs. 24.7%); complete distributions and ISO3 codes are reported in Supplementary Table S5. The coverage matrix, exclusion list, raw API responses, indicator metadata, and processed panel were retained in the reproducibility archive.

### 2.3 Variables

The outcome was life expectancy at birth in years. The exposure was the population-weighted annual mean PM2.5 concentration, scaled so that the regression coefficient represented a 10 μg/m^3^ difference. Time-varying covariates were the natural logarithm of GDP per capita, current health expenditure as a percentage of GDP, and urban population as a percentage of the total population. These variables captured broad economic resources, health-system spending, and urban transition, respectively. They were not assumed to exhaust the confounding structure, and health expenditure or urbanization could partly lie on pathways linking development, emissions, and longevity.

A secondary model included an interaction between scaled PM2.5 and log GDP, centered at the sample median. Because an interaction in a confounded ecological model cannot establish biological susceptibility or effect modification, it was used only to describe how the conventional coefficient varied over the observed development distribution.

### 2.4 Statistical analysis

The conventional adjusted model was:

$${\mathrm{LE}}_{\mathrm{it}}=\beta {\mathrm{PM}}_{\mathrm{it}}+\gamma X_{\mathrm{it}}+\alpha_{i}+\lambda_{t}+\varepsilon_{\mathrm{it}},$$

where LE_it_ was life expectancy for economy i in year t; PM_it_ was PM2.5 divided by 10; X_it_ contained log GDP per capita, health expenditure, and urbanization; αᵢ denoted economy fixed effects; and λ_t_ denoted year fixed effects. Standard errors were clustered by economy. The coefficient β is an ecological within-economy association conditional on common year shocks and measured covariates. It is not a causal estimand without assumptions excluding time-varying confounding, differential outcome trends, and exposure measurement error.

The baseline model omitted time-varying covariates. The unweighted adjusted model was primary because it is a country-panel diagnostic estimand rather than an individual-level effect estimand. It assigns unit weight to each observed economy-year, so economies with longer panels or more within-economy exposure variation can contribute more information, but population size alone does not determine the coefficient. The population-weighted model was secondary because it gives much greater influence to high-population economies and targets a different, population-weighted ecological association. Other sensitivity analyses included economies contributing at least 20 complete years; years 2000–2019; PM2.5 winsorized at the first and 99th percentiles; and PM2.5 lagged 1 or 2 years.

Covariance sensitivity used Bartlett kernel Driscoll–Kraay standard errors with bandwidth three (28, 29). Bandwidth three was used in the originally submitted analysis, is close to T^1/3^, and was retained as the reference bandwidth rather than selected from the reviewer-requested grid. Driscoll–Kraay estimation changes the covariance matrix, not the coefficient or the mean model specification, and therefore cannot correct omitted differential trends. Because the time dimension is short and the primary-model residuals remained serially correlated, the pre-review bandwidth-three analysis is treated as an inference sensitivity check. In response to peer review, a *post hoc* grid recomputed the Driscoll–Kraay covariance estimator at bandwidths 1 through 8; the point estimate and mean specification were held fixed.

Country-specific linear trends allowed each economy to have a separate linear outcome trajectory. First-difference models regressed annual changes in life expectancy on annual changes in exposure and covariates with year fixed effects. Five-year-difference models retained every valid rolling change, defined as the value in year *t* minus that in year t-5, for the outcome, exposure, and covariates. Adjacent 5-year changes therefore overlapped; they were not non-overlapping blocks. Economy-clustered standard errors were used for the change models to allow arbitrary within-economy dependence, including dependence induced by overlapping 5-year windows. These estimators remove persistent level differences and place greater weight on departures from local trajectories, but they can also attenuate slowly varying causal signals or amplify measurement error (30, 31).

The negative-control model replaced current PM2.5 with PM2.5 measured 2 years later. The originally reported 2-year lead was a single temporal diagnostic because it is unambiguously post-outcome, retains more support than longer leads, and is less vulnerable than a one-year lead to simple reporting-year alignment. Because PM2.5 is highly persistent, the lead is not interpreted as independent exposure information or as a calibrated bias correction. In response to peer review, *post hoc* models added separate +1- and +3-year leads and a conditional model containing both current PM2.5 and the +2-year lead. These additions were labeled as reviewer-requested *post hoc* sensitivities and were not substituted for the pre-review +2-year model. All separate-lead and joint models used the primary covariates, economy and year fixed effects, and economy-clustered standard errors. Region-stratified adjusted models were fitted for World Bank regions containing at least 10 economies and 120 observations. North America and South Asia did not meet the economy-count threshold as separate strata; their economy-years remained in the pooled global analysis, but no region-specific coefficient was reported for either region.

Within-country variance inflation factors assessed collinearity among exposure and covariates after economy demeaning. Residual serial dependence was summarized by the lag-one correlation. Ninety-five percent confidence intervals accompanied all coefficients. Sensitivity analyses were interpreted based on direction, magnitude, and interval compatibility rather than a binary significance threshold. Analyses were deterministic and used Python 3.12, pandas 3.0.3, statsmodels 0.14.6, and linearmodels 7.0. Exact commands, package versions, raw responses, checksums, and figure-generation code are archived. OpenAI Codex (GPT-5, OpenAI; accessed on 13 July, 23 August, and 29 August 2026) assisted with literature-metadata verification, implementation and execution of analysis code, figure preparation, manuscript drafting, and language editing; the authors reviewed and verified all outputs.

## 3 Results

### 3.1 Sample and temporal distributions

The mean PM2.5 exposure was 23.973 μg/m^3^ (*N* = 4,406; standard deviation, 15.400), and mean life expectancy was 70.195 years (*N* = 4,406; standard deviation, 8.683). Median PM2.5 declined from 22.895 μg/m^3^ in 2000 to 18.137 μg/m^3^ in 2023, while median life expectancy increased from 70.026 to 73.599 years. Between-economy dispersion was much larger than within-economy change: the within-economy standard deviations were 3.406 μg/m^3^ for PM2.5 and 2.492 years for life expectancy (Table 1). Figure 1 shows that the global distributions moved in generally opposite directions, but the interquartile bands remained wide throughout the study period.

**Table 1:** Characteristics of the complete-case economy-year analysis sample.

Variable	*N*	Mean (SD)	Within-economy SD	Median (IQR)
Life expectancy at birth (years)	4,406	70.20 (8.68)	2.49	71.58 (64.67–76.59)
Annual mean PM2.5 exposure (μg/m^3^)	4,406	23.97 (15.40)	3.41	20.22 (13.58–30.82)
GDP per capita, PPP (constant 2021 international $)	4,406	21855.02 (23571.64)	4325.60	13238.65 (4844.45–30916.42)
Current health expenditure (% of GDP)	4,406	6.23 (2.86)	1.20	5.67 (4.14–7.98)
Urban population (% of total)	4,406	57.16 (22.72)	3.51	58.44 (38.77–74.85)
Population	4,406	37943165.82 (140751809.81)	10557484.83	7315731.50 (1892141.75–25526176.25)

**Figure 1:**
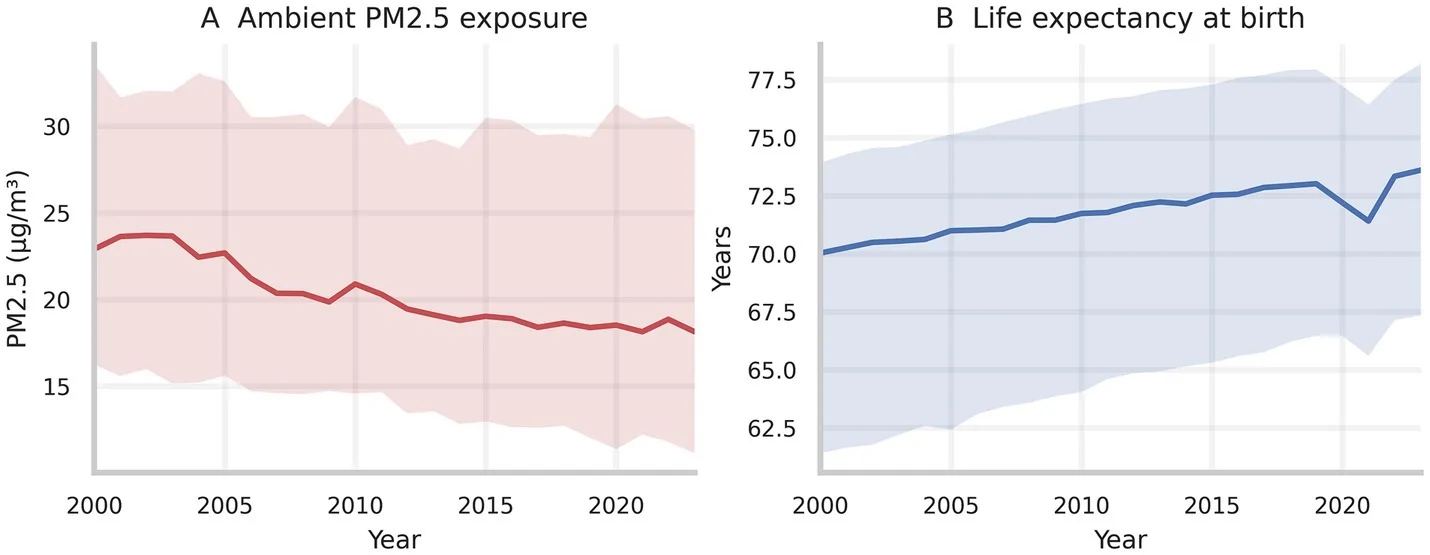
Annual distributions of ambient PM2.5 exposure and life expectancy, 2000–2023. Lines show annual medians among economies in the complete-case analysis sample; shaded bands show interquartile ranges. Panel **A** reports the population-weighted annual mean PM2.5 concentration in μg/m^3^. Panel **B** reports life expectancy at birth in years. The number of economies ranges from 179 in 2000 to 184 in 2023. Source: Authors’ original analysis and visualization of World Bank World Development Indicators data (references 2 and 3), available under the Creative Commons Attribution 4.0 International license (CC BY 4.0).

### 3.2 Conventional fixed-effects estimates

The economy-and-year fixed-effects baseline yielded 0.391 additional years of life expectancy per 10 μg/m^3^ higher PM2.5 (95% confidence interval, 0.107 to 0.674). The primary adjusted estimate was 0.406 years per 10 μg/m^3^ (95% confidence interval, 0.144 to 0.668; *N* = 4,406; *p* = 0.002) after adjustment for log GDP, health expenditure, and urbanization. Figure 2 presents the partialled-out relation underlying this coefficient. The positive sign was opposite to established evidence that greater long-term PM2.5 exposure increases mortality and shortens longevity; it was therefore treated as a sign discrepancy requiring model diagnostics, not as evidence of a protective effect.

**Figure 2:**
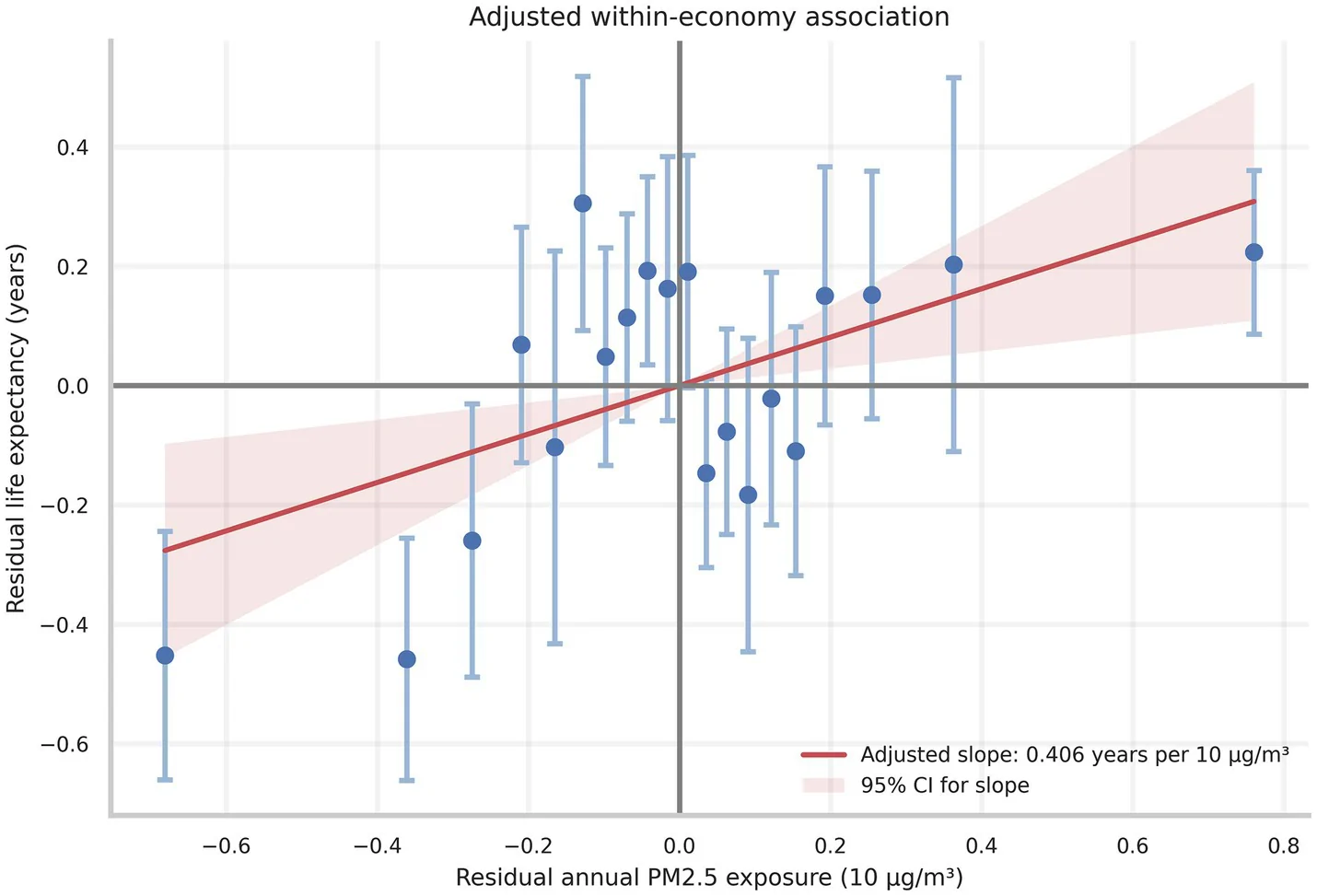
Adjusted within-economy association in the conventional model. PM2.5 and life expectancy were residualized against log GDP per capita, health expenditure, urbanization, economy indicators, and year indicators. Points show the means in 20 equal-frequency bins; vertical bars show 95% confidence intervals for the bin means. The fitted line and shaded band represent the adjusted fixed-effects coefficient and its economy-clustered 95% confidence interval. Residualization is used for visualization only; inference is based on the panel model. Source: Authors’ original analysis and visualization of World Bank World Development Indicators data (references 2 and 3), available under the Creative Commons Attribution 4.0 International license (CC BY 4.0).

Changing the covariance estimator did not explain the conventional result: with Driscoll–Kraay standard errors and bandwidth three, the interval was 0.138 to 0.674 years. Across the full Driscoll–Kraay bandwidth grid from 1 through 8, with the originally reported bandwidth-three result reproduced and the other bandwidths added during peer review, the point estimate remained 0.406 years and every 95% confidence interval excluded zero (largest *p* = 0.006; Supplementary Table S3). Restricting the sample to economies with at least 20 complete years gave 0.413 years (95% confidence interval, 0.150 to 0.675), and restricting the period to 2000–2019 gave 0.275 years (95% confidence interval, 0.019 to 0.532). Winsorizing PM2.5 produced 0.424 years (95% confidence interval, 0.144 to 0.704). The one-year-lag estimate was 0.424 years (95% confidence interval, 0.156 to 0.693; *N* = 4,227), and the two-year-lag estimate was 0.376 years (95% confidence interval, 0.118 to 0.634). The population-weighted estimate was 0.296 years (95% confidence interval, −0.215 to 0.808; *N* = 4,406), with greater uncertainty and compatibility with zero (Table 2). The unweighted and population-weighted models answer different ecological questions; their difference should not be interpreted as a formal test of effect modification by population size.

**Table 2:** PM2.5 coefficient across model specifications. Estimates are years of life expectancy per 10 μg/m^3^ higher exposure.

Specification	Estimate (95% CI)	*N*	Economies	*p* value
Economy and year fixed effects	0.391 (0.107, 0.674)	4,406	186	0.007
Primary adjusted model	0.406 (0.144, 0.668)	4,406	186	0.002
Population-weighted sensitivity model	0.296 (−0.215, 0.808)	4,406	186	0.256
Driscoll-Kraay SE (bandwidth 3)	0.406 (0.138, 0.674)	4,406	186	0.003
At least 20 observed years	0.413 (0.150, 0.675)	4,357	182	0.002
Restricted to 2000–2019	0.275 (0.019, 0.532)	3,665	186	0.036
Economy-specific linear trends	−0.023 (−0.175, 0.129)	4,406	186	0.766
PM2.5 winsorized at 1st/99th percentiles	0.424 (0.144, 0.704)	4,406	186	0.003
PM2.5 lagged 1 year	0.424 (0.156, 0.693)	4,227	186	0.002
PM2.5 lagged 2 years	0.376 (0.118, 0.634)	4,048	186	0.004
Future-exposure negative control (+2 years)	0.296 (0.042, 0.549)	4,037	186	0.022
First differences with year fixed effects	−0.006 (−0.085, 0.073)	4,220	186	0.882
Rolling five-year differences with year fixed effects	0.135 (−0.028, 0.297)	3,476	186	0.105

In the descriptive interaction model, the PM2.5-by-log-GDP coefficient was 0.399 years per 10 μg/m^3^ per one-unit higher log GDP (95% confidence interval, 0.170 to 0.627; *N* = 4,406). The implied conventional PM2.5 coefficient ranged from 0.063 years (95% confidence interval, −0.251 to 0.378) at the 25th percentile of GDP per capita to 0.802 years (95% confidence interval, 0.405 to 1.200) at the 75th percentile. This gradient did not resolve the biological contradiction and was treated as evidence that development trajectories remained embedded in the conventional coefficient (Supplementary Table S2).

### 3.3 Trend, change, and negative-control diagnostics

Specifications that directly altered control for trends markedly attenuated or reversed the positive association (Figure 3). The economy-specific-trend estimate was −0.023 years per 10 μg/m^3^ (95% confidence interval, −0.175 to 0.129; *N* = 4,406). The first-difference estimate was −0.006 years (95% confidence interval, −0.085 to 0.073; *N* = 4,220), and the rolling 5-year-difference estimate was 0.135 years (95% confidence interval, −0.028 to 0.297; *N* = 3,476). The intervals for all three specifications included zero, and their central estimates were markedly smaller than the conventional positive estimate.

**Figure 3:**
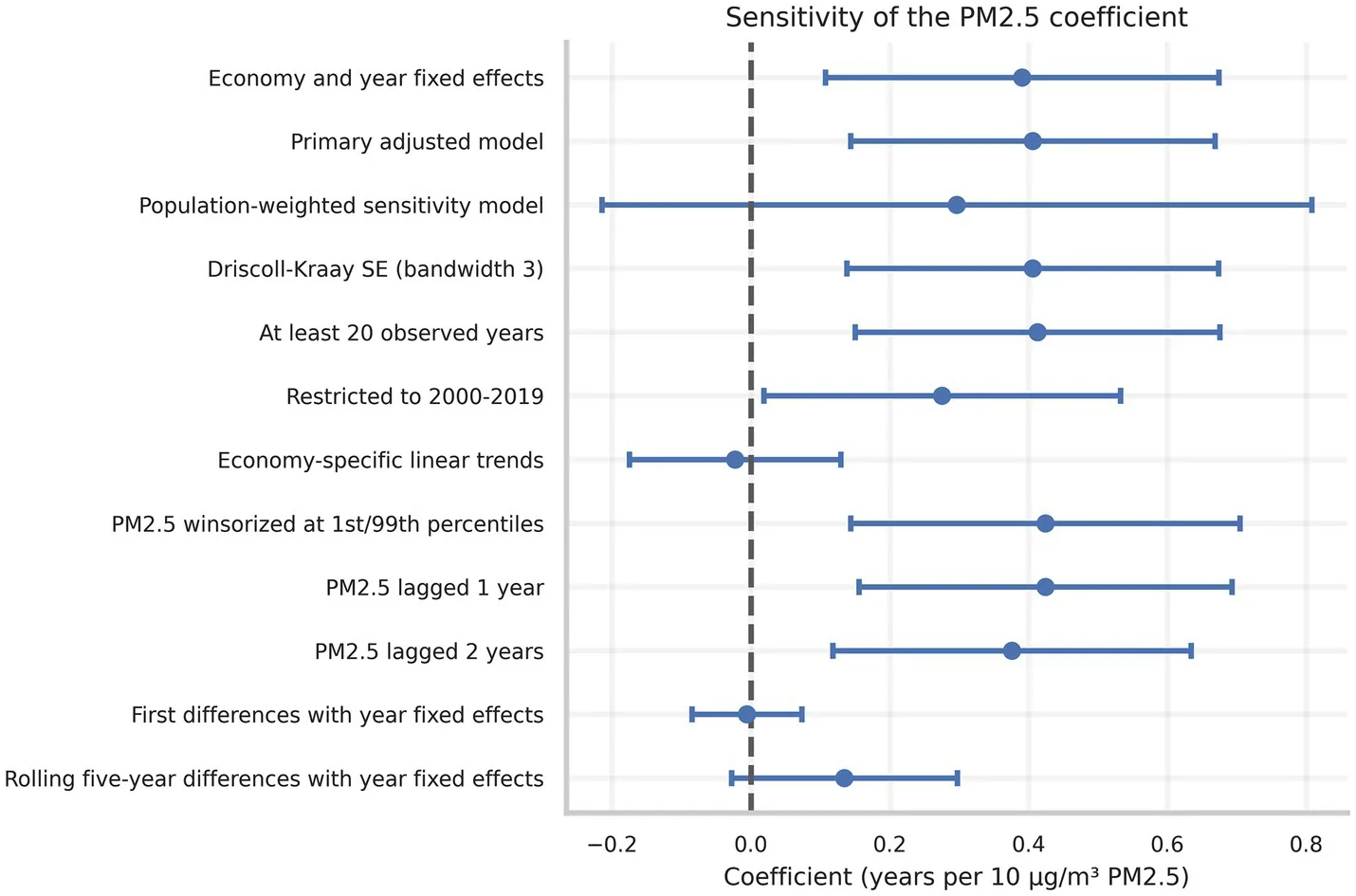
PM2.5 coefficient across model specifications. Points show the estimated change in life expectancy in years per 10 μg/m^3^ higher PM2.5; horizontal bars show 95% confidence intervals. Specifications vary in covariate adjustment, weights, covariance estimator, panel completeness, period, economy-specific trends, exposure treatment, lags, and temporal differencing. The originally reported two-year future-exposure model is reported in Table 2; reviewer-requested covariance-bandwidth and future-lead sensitivities are reported in Supplementary Tables S3, S4. Source: Authors’ original analysis and visualization of World Bank World Development Indicators data (references 2 and 3), available under the Creative Commons Attribution 4.0 International license (CC BY 4.0).

The originally reported separate +2-year future-exposure estimate was 0.296 years per 10 μg/m^3^ (95% confidence interval, 0.042 to 0.549; *N* = 4,037; *p* = 0.022). Thus, PM2.5 measured 2 years after the life-expectancy outcome retained the same positive direction as current and lagged exposure. Because PM2.5 is persistent, this coefficient is neither a separate causal effect nor an independent negative-control exposure. In the expanded temporal profile, which reproduced the original +2-year result and added +1- and +3-year models during peer review, the separate future-exposure estimates attenuated with increasing lead time: +1: 0.369 (0.122 to 0.616); +2: 0.296 (0.042 to 0.549); +3: 0.228 (−0.006 to 0.463) years per 10 μg/m^3^. When current PM2.5 and the +2-year lead were entered together, the future-exposure coefficient was 0.152 years (95% confidence interval, −0.039 to 0.342; *p* = 0.119). The attenuation and imprecision of the conditional estimate make the lead pattern a diagnostic concern about unresolved temporal structure, not a stand-alone demonstration of bias or a corrected health-effect estimate (Supplementary Table S4). The lag-one residual correlation of the primary model was 0.638, consistent with substantial remaining serial structure. Within-country collinearity was limited; the maximum variance inflation factor was 1.388.

### 3.4 Regional heterogeneity

Regional coefficients varied in direction (Figure 4; Supplementary Table S1). The Latin America and Caribbean estimate was −0.510 years per 10 μg/m^3^ (95% confidence interval, −0.841 to −0.179; *N* = 744). Europe and Central Asia had a positive estimate of 0.695 years (95% confidence interval, 0.074 to 1.316; *N* = 1,187). Estimates for East Asia and Pacific, the Middle East and North Africa grouping, and Sub-Saharan Africa had intervals that included zero. North America and South Asia remained in the pooled global analysis but were not reported as separate strata because they did not meet the minimum economy count; no region-specific inference should therefore be drawn for those two regions. The regional contrast did not identify biological effect modification because each model remained ecological and differently confounded. It demonstrated that a single global coefficient was not transportable across the observed regional trajectories.

**Figure 4:**
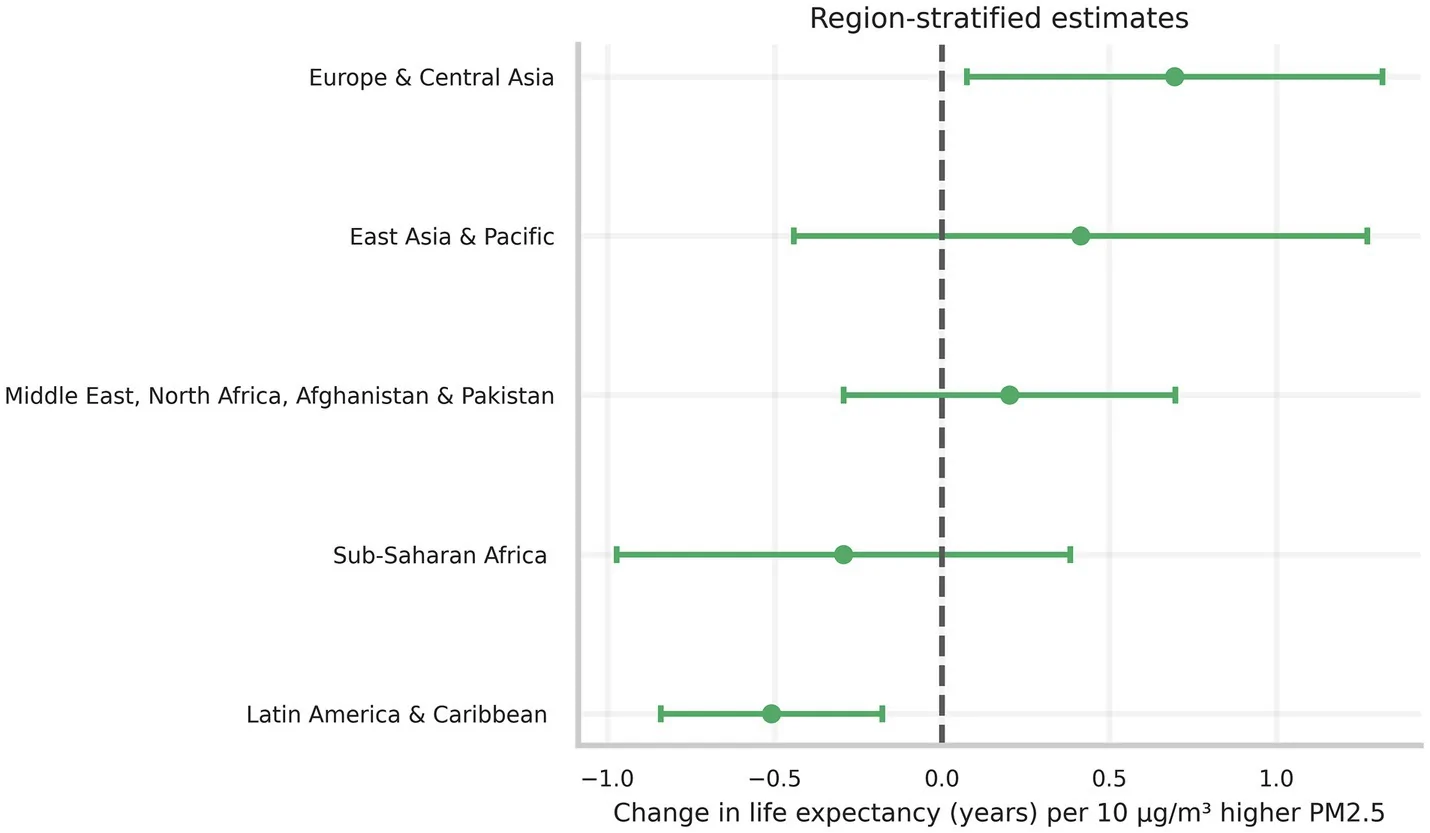
Region-stratified adjusted estimates. Points show adjusted two-way fixed-effects coefficients and horizontal bars show economy-clustered 95% confidence intervals. Regions with fewer than 10 economies or 120 observations were not modeled separately. North America and South Asia remained in the pooled analysis but are not shown as separate strata. Regional contrasts are descriptive and do not identify effect modification. Source: Authors’ original analysis and visualization of World Bank World Development Indicators data (references 2 and 3), available under the Creative Commons Attribution 4.0 International license (CC BY 4.0).

## 4 Discussion

### 4.1 Principal findings

A conventional global country-year model produced a positive association between PM2.5 and life expectancy. That coefficient persisted under alternative standard errors, sample-length restrictions, restriction to pre-2020 years, winsorization, and exposure lags, but it did not persist when the model allowed economy-specific trends or analyzed annual changes. PM2.5 exposure measured in the future was positively associated with earlier life expectancy. Taken together, the sign change, change-model attenuation, and future-lead pattern indicate that the conventional coefficient cannot be interpreted as a protective effect of fine particulate matter or as an estimate of its health effect. The pattern raises concern about differential national trajectories and unresolved serial structure, although the imprecise conditional +2-year lead means that it does not by itself establish that trend heterogeneity was the only source; exposure measurement error, unmeasured shocks, and functional-form misspecification may also contribute.

This conclusion does not weaken the broader evidence that long-term particulate exposure harms health. The 0.406-year positive coefficient has the opposite direction from the US county change estimate reported by Pope and colleagues (5) and from the Huai River quasi-experimental estimate (6). It is also inconsistent with cohort-based mortality syntheses (8, 10–13) and global exposure-response models (7, 9, 22). The discrepancy is informative because it identifies limited credibility of the ecological identifying variation rather than a plausible reversal of particulate toxicity.

### 4.2 Why the conventional coefficient reversed direction

Country fixed effects remove stable differences among economies, and year fixed effects remove shocks common to all economies. Neither component removes an economy’s own development path. From 2000 to 2023, many economies experienced simultaneous changes in income, health care, infectious-disease control, education, urbanization, energy use, emissions, and mortality. These processes need not have common slopes. If life expectancy improves rapidly during periods when industrialization or population-weighted pollution also rises, the residual within-economy association can be positive even when particulate exposure is harmful at the individual level. Conversely, economies with established pollution controls may experience declining PM2.5 along a slower, already high life-expectancy trajectory. A common year effect does not reconcile those heterogeneous paths.

The empirical diagnostics are consistent with this explanation. Adding a separate linear trend for each economy moved the coefficient from 0.406 to −0.023 years. Annual differencing moved it to −0.006 years, and rolling five-year differencing produced a smaller, imprecise positive coefficient. The separate future-exposure estimates attenuated from +1 through +3 years, and the +2-year coefficient was smaller and imprecise when current exposure was included. This pattern is compatible with persistent trajectories but does not identify a unique bias mechanism. Ma and colleagues similarly found that fixed-effect selection materially altered PM2.5-hospitalization estimates across 52 models (17). Imai and Kim (18) and Arkhangelsky and Imbens (19) formalize why two-way fixed effects require stronger assumptions than the presence of unit and time indicators alone. These diagnostics do not isolate one unique bias mechanism, but they show that the conventional coefficient depends on low-frequency variation that the aggregate model cannot cleanly separate from heterogeneous development and mortality trajectories.

Aggregation adds further difficulty. National population-weighted PM2.5 is not personal exposure and cannot represent within-country environmental inequality, source composition, occupation, indoor infiltration, or mobility. Life expectancy integrates mortality from communicable diseases, non-communicable diseases, injuries, conflict, and health-system disruption. A national GDP, health-expenditure share, and urbanization percentage cannot control all joint determinants of these two aggregates. The stronger positive coefficient at higher GDP levels is therefore not evidence that PM2.5 becomes beneficial with development. It may instead reflect the extent to which the conventional interaction inherited heterogeneous economic and mortality trajectories.

### 4.3 Relation to prior country and subnational panels

Previous aggregate analyses have often reported the expected negative relation between air pollution and life expectancy (14, 15). Differences from the present result can arise from geography, period, pollution indicator, covariate choice, weighting, dynamic specification, and the balance between cross-sectional and within-unit information. The present analysis did not attempt to select the specification that best reproduced the expected sign. It retained the conventional positive estimate and evaluated whether it survived tests directed at its temporal identifying assumptions.

That approach also clarifies the role of apparently contradictory studies. Cox (16) questioned constant causal interpretations of life-expectancy regressions and emphasized non-linearity and temporal ambiguity. Subsequent analyses supported the stability of the original US association to influential observations, yet that debate occurred in a setting with two multi-year contrasts and more local exposure measures. The global WDI panel has broader coverage but coarser variables and far more heterogeneous data-generating processes. Larger geographic scope does not compensate for weaker exchangeability.

### 4.4 Implications for environmental health analysis

Country-year panels remain useful for surveillance, descriptive comparisons, hypothesis generation, and identification of data inconsistencies. Their coefficients require a diagnostic sequence before health-effect interpretation. At minimum, a study should show exposure and outcome trajectories, quantify within-unit variation, define the unweighted or weighted estimand, justify the fixed-effect structure, examine unit-specific trends or flexible latent factors, compare level and change estimators, report covariance sensitivity across defensible bandwidths, test prespecified exposure lags and leads, and use negative controls where temporal ordering permits. A specification matrix should be interpreted by estimand and assumption rather than by the number of coefficients below a *p*-value threshold.

When the scientific objective is causal, stronger designs are preferable. Quasi-experiments based on regulatory discontinuities, policy shocks, wind direction, or other exogenous exposure variation can separate pollution changes from concurrent development, provided their own assumptions are credible. Individual or small-area cohorts can align exposure, confounders, and outcomes more closely, although they also require careful control of spatial and temporal confounding. Harmonized multi-pollutant studies should examine PM2.5 together with NO2, ozone, source composition, and within-country exposure inequality rather than assuming that one national indicator represents the full pollution mixture. Global aggregate models can complement these designs by describing coverage and heterogeneity, but they should not overwrite better-identified evidence when their trend or temporal diagnostics raise unresolved concerns.

The future-exposure diagnostic was informative but not definitive. Future exposure is not an ideal negative control when exposure is persistent: anticipation, reporting alignment, measurement smoothing, or long disease latency can complicate its magnitude and interpretation. Life expectancy in year *t* cannot be caused by ambient exposure measured in year *t* + 2, so a positive separate-lead coefficient raises concern about unresolved temporal structure. However, the attenuation across +1 to +3 years and the imprecise +2-year coefficient after adjustment for current PM2.5 show why the lead pattern should not be described as conclusive falsification. Negative controls are diagnostic tools and do not identify a corrected effect (20, 21).

### 4.5 Strengths and limitations

The analysis used the July 2026 WDI release and matched data through 2023, preserved every raw API response, froze a content-addressed analysis sample, and generated all tables and figures from code. The panel covered 186 economies and 4,406 economy-years. The specification set included conventional, weighted, lagged, trend-adjusted, change-based, regional, and negative-control analyses. Null results and the unexpected positive coefficient were retained, which allowed the study to address model credibility rather than reproduce a preferred direction.

Several limitations bound the conclusions. First, the design is ecological. No coefficient can be assigned to individuals, and national aggregation can induce ecological bias. Second, the analysis focused on PM2.5 and did not estimate independent or joint effects of NO2, ozone, or particle source composition. National PM2.5 exposure combines satellite observations, models, monitors, and population weights; measurement error and changes in monitoring inputs may be temporally structured. Third, life expectancy is a composite period measure with no cause-specific decomposition in this dataset. Fourth, smoking, education, inequality, conflict, epidemic intensity, health-service quality, migration, and policy were not comprehensively measured. Adding available macro covariates would not guarantee control of their measurement error or time-varying effects.

Fifth, country-specific linear trends can absorb slowly varying causal exposure signals as well as confounding, while first differences can amplify measurement noise (30, 31). Their null estimates are therefore not estimates of no harm; they show that the positive conventional coefficient depends on low-frequency variation that the aggregate data cannot separate from differential trends. Sixth, the COVID-19 pandemic altered mortality, mobility, health care, and emissions. A pre-2020 restriction reduced but did not reverse the positive conventional coefficient; this sensitivity analysis addresses pandemic disruption without denying evidence linking long-term PM2.5 with COVID-19 mortality (24–26). Seventh, complete-case eligibility excluded 31 economies or territories. Their income-group and regional composition differed descriptively from that of the 186 included economies, and the missingness mechanism was not modeled (27). This pattern may limit generalizability, and the analytic panel should not be interpreted as population-representative (Supplementary Table S5). Regional models also had unequal sample sizes, and North America and South Asia could not be estimated as separate strata under the defined analysis threshold. Finally, no instrument, policy discontinuity, external individual-level cohort, or cause-specific outcome was available to identify a causal effect. The study consequently supports a model-diagnostic conclusion rather than a quantitative health-effect estimate.

## 5 Conclusion

In a global panel covering 2000–2023, a conventional model with economy and year fixed-effects produced a positive PM2.5-life-expectancy coefficient. That coefficient was highly sensitive to how low-frequency trends were controlled. Separate future-exposure estimates remained positive but attenuated across +1 to +3 years, and the +2-year lead was smaller and imprecise after adjustment for current exposure. These findings raise concern about unresolved differential national trajectories and serial structure; they do not prove that differential trends are the sole cause and do not estimate either benefit or harm from PM2.5. Future work should prespecify richer distributed lead–lag profiles, evaluate harmonized multi-pollutant PM2.5-NO2-ozone models, characterize missing-data selection, and prioritize quasi-experimental or finer-scale designs that align exposure, confounding, and mortality. Global ecological panels can remain useful for surveillance and hypothesis generation, but their coefficients should inform health-effect interpretation only when trend and falsification assumptions are credible.

## Data Availability

Publicly available datasets were analyzed in this study. This data can be found here: Zenodo v4: https://doi.org/10.5281/zenodo.22150226 (concept DOI https://doi.org/10.5281/zenodo.21339626).
