# Supplementary material for: Paradoxical PM2.5-longevity estimates in global country panels are sensitive to differential trend control: a specification analysis, 2000–2023
Source: Front Public Health. 2026 Oct 1;14:1936760. doi: 10.3389/fpubh.2026.1936760 (PMC13669107; doi:10.3389/fpubh.2026.1936760)
Supplement: Supplementary file 1 [file Data_Sheet_1.PDF]

Supplementary Material

This file contains Supplementary Tables S1-S5. Tables S3 and S4 combine reproduced original estimates with reviewer-requested post hoc sensitivities; each row is classified accordingly. Table S5 reports the quantitative selection summary added during peer review.

1 Supplementary Tables

Supplementary Table S1. Region-stratified adjusted two-way fixed-effects estimates.

World Bank region	Estimate	95% CI	p value	N	No. economies
East Asia & Pacific	0.414	-0.443 to 1.271	0.343	693	29
Europe & Central Asia	0.695	0.074 to 1.316	0.028	1187	50
Latin America & Caribbean	-0.510	-0.841 to -0.179	0.003	744	31
Middle East, North Africa, Afghanistan & Pakistan	0.202	-0.294 to 0.697	0.424	509	22
Sub-Saharan Africa	-0.295	-0.972 to 0.383	0.394	1081	46

Note. Estimates are years of life expectancy per 10 µg/m³ higher PM2.5. Models include economy and year fixed effects and adjust for log GDP per capita, health expenditure, and urbanization. Standard errors are clustered by economy.

Supplementary Table S2. PM2.5-by-income interaction and income-specific marginal estimates.

Estimate type	GDP quantile	GDP per capita	Estimate	95% CI	p value	N
PM2.5-by-log-GDP interaction			0.399	0.170 to 0.627	<0.001	4406
PM2.5 marginal effect	0.25	4844.45	0.063	-0.251 to 0.378		4406
PM2.5 marginal effect	0.50	13238.65	0.464	0.170 to 0.758		4406
PM2.5 marginal effect	0.75	30916.42	0.802	0.405 to 1.200		4406

Note. GDP per capita is purchasing-power-parity adjusted in constant 2021 international dollars. Estimates are descriptive ecological contrasts and do not identify biological effect modification.

Supplementary Table S3. Driscoll-Kraay bandwidth sensitivity profile with reviewer-requested additions.

BW	β	SE	95% CI	p	N	Econ.	Status
1	0.406	0.115	0.181 to 0.631	<0.001	4406	186	Reviewer-requested post hoc
2	0.406	0.127	0.156 to 0.656	0.001	4406	186	Reviewer-requested post hoc
3	0.406	0.137	0.138 to 0.674	0.003	4406	186	Original analysis reproduced
4	0.406	0.142	0.128 to 0.684	0.004	4406	186	Reviewer-requested post hoc
5	0.406	0.145	0.121 to 0.691	0.005	4406	186	Reviewer-requested post hoc
6	0.406	0.147	0.118 to 0.694	0.006	4406	186	Reviewer-requested post hoc
7	0.406	0.146	0.121 to 0.691	0.005	4406	186	Reviewer-requested post hoc
8	0.406	0.144	0.124 to 0.687	0.005	4406	186	Reviewer-requested post hoc

Note. The mean specification and point estimate are identical across rows. Only the Bartlett-kernel Driscoll-Kraay covariance bandwidth changes. Bandwidth three reproduces the originally reported sensitivity; all other bandwidths were added during peer review.

Supplementary Table S4. Future-exposure temporal profile with reviewer-requested additions.

Specification	Term	Time	Model	β	95% CI	p	N	Status
PM2.5 lag 2 years	PM2.5 lag 2	t-2	Single	0.376	0.118 to 0.634	0.004	4048	Original analysis reproduced
PM2.5 lag 1 year	PM2.5 lag 1	t-1	Single	0.424	0.156 to 0.693	0.002	4227	Original analysis reproduced
Current PM2.5 exposure	PM2.5	t	Single	0.406	0.144 to 0.668	0.002	4406	Original analysis reproduced
Future-exposure negative control (+1 year)	PM2.5 lead +1	t+1	Single	0.369	0.122 to 0.616	0.003	4222	Reviewer-requested post hoc
Future-exposure negative control (+2 years)	PM2.5 lead +2	t+2	Single	0.296	0.042 to 0.549	0.022	4037	Original analysis reproduced

Supplementary Material

Specification	Term	Time	Model	β	95% CI	p	N	Status
Future-exposure negative control (+3 years)	PM2.5 lead +3	t+3	Single	0.228	-0.006 to 0.463	0.056	3851	Reviewer-requested post hoc
Current and +2-year future PM2.5 jointly	Current PM2.5	t	Joint	0.275	0.077 to 0.473	0.007	4037	Reviewer-requested post hoc
Current and +2-year future PM2.5 jointly	+2-year PM2.5	t+2	Joint	0.152	-0.039 to 0.342	0.119	4037	Reviewer-requested post hoc

Note. The self-contained temporal profile reproduces the original lag-2, lag-1, current, and separate lead-2 estimates and adds reviewer-requested separate lead-1 and lead-3 estimates. The joint model contributes two rows: the current-exposure and lead-2 coefficients estimated simultaneously. Stand-alone leads are not independent causal paths because annual PM2.5 is persistent. The added analyses use the primary covariates, economy and year fixed effects, and economy-clustered standard errors. They are diagnostic and do not estimate a corrected causal effect.

Supplementary Table S5. Distribution of the 31 economies excluded by complete-case eligibility.

Group	Category	n	%	ISO3 codes
Region	Latin America & Caribbean	11	35.5%	ABW;CUB;CUW;CYM;MAF;PRI;SXM;TCA;VEN;VGB;VIR
Region	East Asia & Pacific	8	25.8%	ASM;GUM;HKG;MAC;MNP;NCL;PRK;PYF
Region	Europe & Central Asia	8	25.8%	CHI;FRO;GIB;GRL;IMN;LIE;MCO;XKX
Region	Sub-Saharan Africa	2	6.5%	ERI;SSD
Region	Middle East, North Africa, Afghanistan & Pakistan	1	3.2%	YEM
Region	North America	1	3.2%	BMU
Income group	High income	24	77.4%	ABW;ASM;BMU;CHI;CUW;CYM;FRO;GIB;GRL;GUM;HKG;IMN;LIE;MAC;MAF;MCO;MNP;NCL;PRI;PYF;SXM;TCA;VGB;VIR
Income group	Low income	4	12.9%	ERI;PRK;SSD;YEM
Income group	Upper middle income	2	6.5%	CUB;XKX
Income group	Lower middle income	1	3.2%	VEN

Note. Counts are descriptive and use the World Bank classifications retained with the coverage audit. Percentages within each dimension sum to 100% apart from rounding. For comparison, among the 186 included economies, 62 (33.3%) were high income; 31 (16.7%) were in Latin America & Caribbean; 29 (15.6%) were in East Asia & Pacific; and 46 (24.7%) were in Sub-Saharan Africa. Comparisons are descriptive and do not constitute a test of the missingness mechanism.
